# Deficiency in plasmacytoid dendritic cells and type I interferon signalling prevents diet-induced obesity and insulin resistance in mice

**DOI:** 10.1007/s00125-017-4341-0

**Published:** 2017-06-28

**Authors:** Tine D. Hannibal, Anja Schmidt-Christensen, Julia Nilsson, Nina Fransén-Pettersson, Lisbeth Hansen, Dan Holmberg

**Affiliations:** 1 Department of Experimental Medical Sciences, Lund University, Biomedical Center, CRC, 205 02 Malmö, Sweden; 2 Department of Immunology & Microbiology, Faculty of Health and Medical Sciences, University of Copenhagen, Copenhagen, Denmark

**Keywords:** Adipose tissue, Obesity, pDCs, Type I interferons

## Abstract

**Aims/hypothesis:**

Obesity is associated with glucose intolerance and insulin resistance and is closely linked to the increasing prevalence of type 2 diabetes. In mouse models of diet-induced obesity (DIO) and type 2 diabetes, an increased fat intake results in adipose tissue expansion and the secretion of proinflammatory cytokines. The innate immune system not only plays a crucial role in obesity-associated chronic low-grade inflammation but it is also proposed to play a role in modulating energy metabolism. However, little is known about how the modulation of metabolism by the immune system may promote increased adiposity in the early stages of increased dietary intake. Here we aimed to define the role of type I IFNs in DIO and insulin resistance.

**Methods:**

Mice lacking the receptor for IFN-α (IFNAR^−/−^) and deficient in plasmacytoid dendritic cells (pDCs) (B6.E2-2^*fl*/*fl*^.Itgax-cre) were fed a diet with a high fat content or normal chow. The mice were analysed in vivo and in vitro using cellular, biochemical and molecular approaches.

**Results:**

We found that the development of obesity was inhibited by an inability to respond to type I IFNs. Furthermore, the development of obesity and insulin resistance in this model was associated with pDC recruitment to the fatty tissues and liver of obese mice (a 4.3-fold and 2.7-fold increase, respectively). Finally, we demonstrated that the depletion of pDCs protects mice from DIO and from developing obesity-associated metabolic complications.

**Conclusions/interpretation:**

Our results provide genetic evidence that pDCs, via type I IFNs, regulate energy metabolism and promote the development of obesity.

## Introduction

Obesity is recognised as a medical condition characterised by the accumulation of excess body fat and is associated with a chronic state of low-grade inflammation. Low-grade inflammation in the adipose tissue (AT) involving both the adaptive and the innate immune system is reflected by a cytokine-induced acute-phase response, including elevated levels of TNF-α, IL-6 and C-reactive protein as part of an immune response of both innate and adaptive origin. A key role in the development of metabolic abnormalities has been assigned to M1-polarised macrophages that accumulate in the AT together with other immune cells [1–3].

A role for dendritic cells (DCs) in promoting macrophage infiltration to AT in obesity has been suggested [4], and an increased frequency of dysfunctional DCs in obese mice and humans supports this notion [4–6]. However, a full understanding of the role of DCs in this process is still lacking.

The Toll-like receptor (TLR)-4 (expressed on the surface of macrophages, DCs and other immune and non-immune cells) has been reported to respond to the increased levels of endogenous lipids found in obese individuals [7]. In line with this, lipid-lowering agents acting through a TLR-4-mediated mechanism can reduce the production of or the responsiveness to IFN-β and reduce the proinflammatory actions of AT macrophages [8]. IFN-β is a member of the type I IFN family of pleiotropic cytokines that is critical in the defence against viral infections and able to modulate both innate and adaptive immunity [9]. IFN-α/β can exert their effects by binding to their cognate receptor, the type I IFN receptor (IFNAR) complex, which stimulates the Janus kinase (JAK) signal transducer and activator of transcription (STAT) signalling pathway, leading to the transcription of several IFN-stimulated genes [10]. While antibody-mediated neutralisation of IFN-β leads to a decrease in the mRNA expression of proinflammatory genes, including monocyte chemoattractant protein (MCP)-1, inducible nitric oxide synthase and IL-6, recombinant IFN-β protein has the opposite effect [11]. In addition, IFN-β has been reported to be necessary for maintaining the TLR-mediated MCP-1 production in macrophages [12], facilitating the recruitment of macrophages to sites of inflammation [13] and modulating the inflammatory and metabolic effects of diet-induced obesity (DIO) [14]. Thus, IFN-β induces a feedback activation mechanism, thereby contributing to macrophage-mediated inflammatory responses.

Type I IFNs are constitutively expressed in low quantities by many tissues and cells of the body in the absence of viral infection [15], supporting a potential physiological role of IFN-α/β as the initial primers of immune function as a means of maintaining immune homeostasis. A role has been proposed for type I IFN in the activation of resident macrophages and the recruitment of proinflammatory M1 macrophages to the AT and liver during obesity.

Plasmacytoid DCs (pDCs) are a unique immune cell population involved in both innate and adaptive immunity. They sense single-stranded RNA and microbial DNA through endosomal TLR-7 and TLR-9, respectively, initiating a myeloid differentiation protein 88 (MyD88)-dependent signalling cascade [16], leading to downstream activation of IRF7 and robust type I IFN responses. The pDCs are responsible for the vast majority of secreted type I IFN that promotes DC maturation, natural killer cell-mediated cytotoxicity and Th1 differentiation, and plays a crucial role in protection against viral and bacterial threats [16]. The induction and subsequent nuclear relocation of the transcription factor IRF-7 is essential for type I IFN expression in pDCs [17]. In comparison, DCs and macrophages from IRF-7 knockout mice were still able to produce normal levels of IFN-β [18].

Studies have shown that IRF-7 deficiency prevents DIO and insulin resistance in mice [19], and that IRF-7 expression is upregulated in the arteries of obese rats [20]. In addition, murine gene expression analyses have reported significant upregulation of IFN-α/β genes in both the visceral and subcutaneous AT during obesity [21], suggesting a role for pDC-derived type I IFN in obesity development. In support of this, elevated frequencies of pDCs were observed in the liver and AT of obese mice [4] and humans [22].

Here, we investigated whether DIO and the associated metabolic abnormalities are dependent on type I IFN signalling. We also analysed pDC, the major type I IFN-producing cellular subset, activity accumulated in the liver and AT during DIO and the effects of induced pDC deficiency on the development of obesity and metabolic abnormalities. We hypothesised that pDCs, through their IFN-producing capacity, not only play significant roles in obesity-induced low-grade inflammation and type 2 diabetes development but also in the early stages of obesity development.

## Methods

### Animals

C57BL/6 (B6) male mice were purchased from Taconic (Ejby, Denmark). IFNAR^−/−^ mice were kindly provided by B. Johansson-Lindbom, Lund University (Lund, Sweden). B6.E2-2^*fl*/*fl*^ mice were generated as previously detailed [23] and B6.Cg-Tg(Itgax-cre)1-1Reiz/J mice were purchased from Jackson Laboratory (Bar Harbor, ME, USA). B6.E2-2^*fl*/*fl*^ and B6.Cg-Tg(Itgax-cre)1-1Reiz/J mice were paired; the offspring were screened for flox-sites and cre-recombinase and were bred to produce the B6.E2-2^*fl*/*fl*^Itgax.cre^+^ and B6.E2-2^*fl*/*fl*^Itgax.cre^−^ mice used in this study.

B6.E2-2^*fl*/*fl*^Itgax.cre mice were bred in a specific pathogen-free animal facility at Lund University and kept under standard conditions with ad libitum access to water and food during experiments. The experiments were conducted in compliance with the National Institutes of Health guidelines, and all animal experimental procedures were approved by the ethical committee of Lund University Animal Care and Use.

### Metabolic effects of DIO

The mice were maintained on either a high-fat diet (HFD; 21.9 kJ/g [5.24 kcal/g], 34.9% (wt/wt) fat, 26.2% (wt/wt) protein, 26.2% (wt/wt) carbohydrate; D12492, Research Diets, New Brunswick, NJ, USA) or a normal diet (ND; 12.6 kJ/g [3 kcal/g], 4% (wt/wt) fat, 18.5% (wt/wt) protein, 55.7% (wt/wt) carbohydrate; R36, Lactamin AB, Stockholm, Sweden) for 18 weeks, starting at 5 weeks of age.

Body weight was recorded weekly. For endpoint analysis, the animals were fasted for 6 h before body composition scanning using a GE-Lunar PIXImus 2 scanner (GE Healthcare, Wauwatosa, WI, USA) or an OGTT, where blood glucose was measured at various time points before and after oral glucose administration (2 g/kg). Cheek blood samples were collected before and after glucose administration for hormone analysis. The mice were anaesthetised with Hypnorm/Dormicum (Vetapharma, Leeds, UK/Roche, Basel, Switzerland) and transcardially perfused with 15 ml PBS, followed by dissection of subcutaneous white adipose tissue (SAT), visceral white adipose tissue (VAT), brown adipose tissue (BAT), the spleen and the liver for flow cytometry.

### Flow cytometric analysis

Single cell suspensions from the spleen were obtained by disrupting the tissue through a 70 μm cell strainer. The livers were mechanically minced into small >1 mm pieces, washed twice in PBS, treated with collagenase II (Invitrogen, Carlsbad, CA, USA) for 40 min at 37°C, and single cells were suspended through a 70 μm cell strainer and separated by 50/30 Percoll (GE Healthcare) gradient centrifugation. The white adipose tissue samples were mechanically minced and collagenase II digestion was performed for 40 min at 37°C to fractionate the AT into adipocytes and a stromal vascular fraction (SVF). The SVF was resuspended in HEPES-buffered RPMI medium (Gibco, Waltham, MA, USA) and single cells were suspended through a 70 μm cell strainer. All samples were resuspended in FACS buffer (PBS, 3% [vol./vol.] FCS, 2 mmol/l EDTA) prior to surface staining, preincubated with anti-CD16/32 (Fc ‘blocking’ antibody, clone 2.4G2) (BD Biosciences, San Jose, CA, USA) for 15 min at 4°C and subsequently stained with fluorescent-labelled primary antibodies for 25 min at 4°C. The following antibodies were used: anti-TCRβ (clone H57-597) and anti-SiglecF (clone E50-2440) from BD Biosciences; anti-CD317 (clone 120G8.04) from Dendritics (Lyon, France); anti-Ly6G (clone 1A8), anti-Ly6C (clone HK1.4), anti-NK1.1 (clone PK136), anti-CD45 (clone 30-F11), anti-I-A/I-E (clone M5/114.15.2), anti-CD11b (clone M1/70) and anti-CD206 (clone C068C2) from Biolegend (San Diego, CA, USA); and anti-CD4 (clone GK1.5), anti-F4/80 (clone BM8), anti-CD45R(B220) (clone RA3-6B2), anti-CD8 (clone 53-6.7), anti-CD11c (clone N418), anti-SiglecH (clone 440c), anti-CD19 (clone 1D3) and fixable viability dye (FVD) from eBioscience (Santa Clara, CA, USA). The cells were fixed using the Foxp3 Staining Buffer Set (eBioscience). Samples containing biotin-coupled antibodies were incubated for 25 min at 4°C with secondary Streptavidin-PECF594 antibodies (BD Biosciences). Cells were washed twice in FACS buffer and data acquisition was performed on a BD LSR II flow cytometer with FACSDiva software (BD Biosciences). A minimum of 50,000 CD45^+^ events were analysed for each sample. The analysis was performed using the FlowJo software (FlowJo LLC, Ashland, OR, USA).

### Insulin ELISA

Cheek blood samples were collected from mice as a part of the OGTT, and plasma was isolated with the aid of EDTA-K tubes (Sarstedt, Nümbrecht, Germany). The tubes were spun at 1000× *g* for 15 min to isolate plasma. Insulin levels were analysed using an Ultra Sensitive Mouse Insulin ELISA kit (CrystalChem, Downers Grove, IL, USA).

### Statistical analysis

Pairwise group comparison was performed using unpaired two-tailed Student’s *t* test or two-way ANOVA using GraphPad Prism 5 (GraphPad Software, San Diego, CA, USA). The values are presented as the mean ± SD.

## Results

### Development of DIO and insulin resistance is dependent on type I IFN signalling

**Fig. 1:**
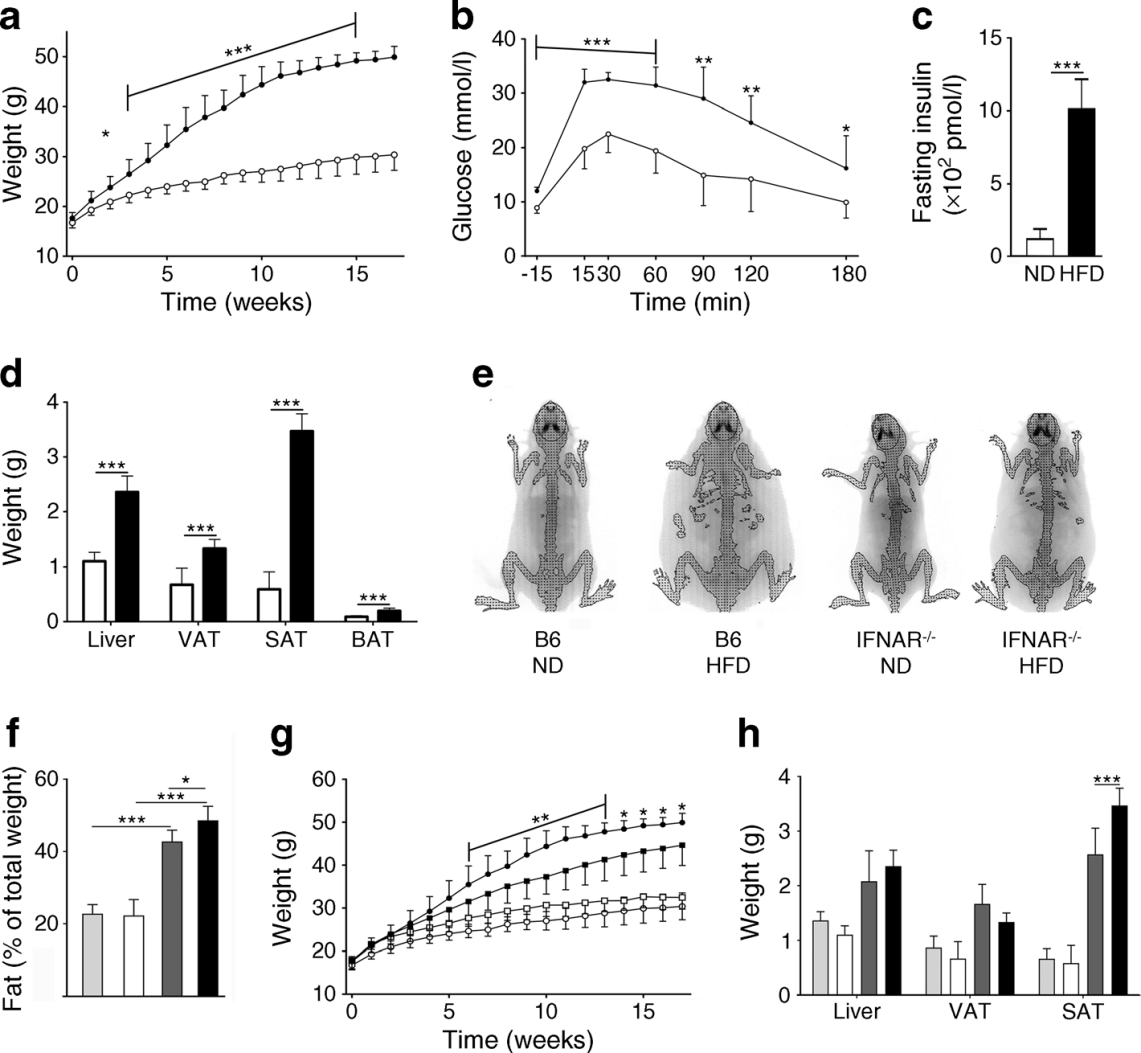
Characteristics and metabolic profile of B6 and IFNAR^−/−^ male mice exposed to ND or HFD. Mice were given an ND or HFD for 18 weeks, starting at 5 weeks of age. (**a**) Weekly weight measurements of the B6 mice fed an ND (white circles) or HFD (black circles). (**b**) An OGTT was performed after 18 weeks. B6 mice were given an oral dose of glucose and blood glucose was determined from tail vein samples at different time points. ND, white circles; HFD, black circles. (**c**) Fasting insulin levels in ND- or HFD-fed B6 mice. (**d**) Individual tissue weights from B6 mice after 18 weeks. ND, white bars; HFD, black bars. (**e**) Image of an x-ray scan determining the fat content in B6 and IFNAR^−/−^ mice after 18 weeks on an ND or HFD. (**f**) Proportion of fat in B6 and IFNAR^−/−^ mice after 18 weeks of an ND or HFD determined by x-ray scanning. (**g**) Weekly weight measurements of the IFNAR^−/−^ (squares) and control B6 (circles) mice fed an ND (white symbols) or HFD (black symbols). A significant difference was only indicated for B6 mice on an HFD. (**h**) Individual tissue weights of IFNAR^−/−^ and B6 mice after 18 weeks. (**f**, **h**) IFNAR^−/−^ ND, light grey bars; IFNAR^−/−^ HFD, dark grey bars; B6 ND, white bars; B6 HFD, black bars. *n* = 6 for ND- and *n* = 18 for HFD-fed animals, except for in (**b**, **c**) where *n* = 6 for ND- and HFD-fed animals. The experiments were repeated independently 2–3 times. *p ≤ 0.05, **p ≤ 0.01, ***p ≤ 0.001 using two-way ANOVA (**a**, **b**, **f**, **g**, **h**) or Student’s *t* test (**c**, **d**)

To gain insight into the potential role of type I IFN in obesity and obesity-related metabolic abnormalities, we used the HFD mouse model of type 2 diabetes. From 5 weeks of age, male C57BL/6 (B6) mice were given ad libitum access to either an HFD or ND for 18 weeks. As shown before [24], mice fed an HFD showed a significant weight increase of 64% compared with ND-fed littermates (Fig. 1a). At the 18 week endpoint, the obese mice had developed glucose intolerance and insulin resistance (Fig. 1b, c). The detrimental stage of metabolic disease was evident by the increase in the sizes of the fat pads and the liver, which were significantly enlarged in obese mice (HFD, 2.35 ± 0.29 g; ND, 1.10 ± 0.16 g) (Fig. 1d). Using an x-ray densitometer, lean and fat mass was compared between the two groups, by which the difference in weight gain was attributed to a significant increase in fat mass (Fig. 1e, f).

To directly test the hypothesis that type I IFN plays a significant role in the development of obesity and type 2 diabetes-related metabolic abnormalities, we exposed mice lacking the receptor for IFN-α (IFNAR^−/−^) to an HFD. In support of the hypothesis, we found that the HFD-fed IFNAR^−/−^ mice displayed a relative resistance to diet-induced weight gain compared with the B6 control mice on an HFD (Fig. 1g). This difference was significant after 6 weeks following the initiation of HFD feeding. At the endpoint after 18 weeks of HFD, the IFNAR^−/−^ mice had an 11% lower body weight compared with the control B6 mice (IFNAR^−/−^HFD 44.7 g vs B6 HFD 49.9 g). This was associated with significantly smaller subcutaneous fat depots and a lower total fat mass in the HFD-fed IFNAR^−/−^ mice compared with B6 mice (Fig. 1f, h).

### DIO led to elevated numbers of proinflammatory macrophages in AT which are reduced in absence of IFNAR1

**Fig. 2:**
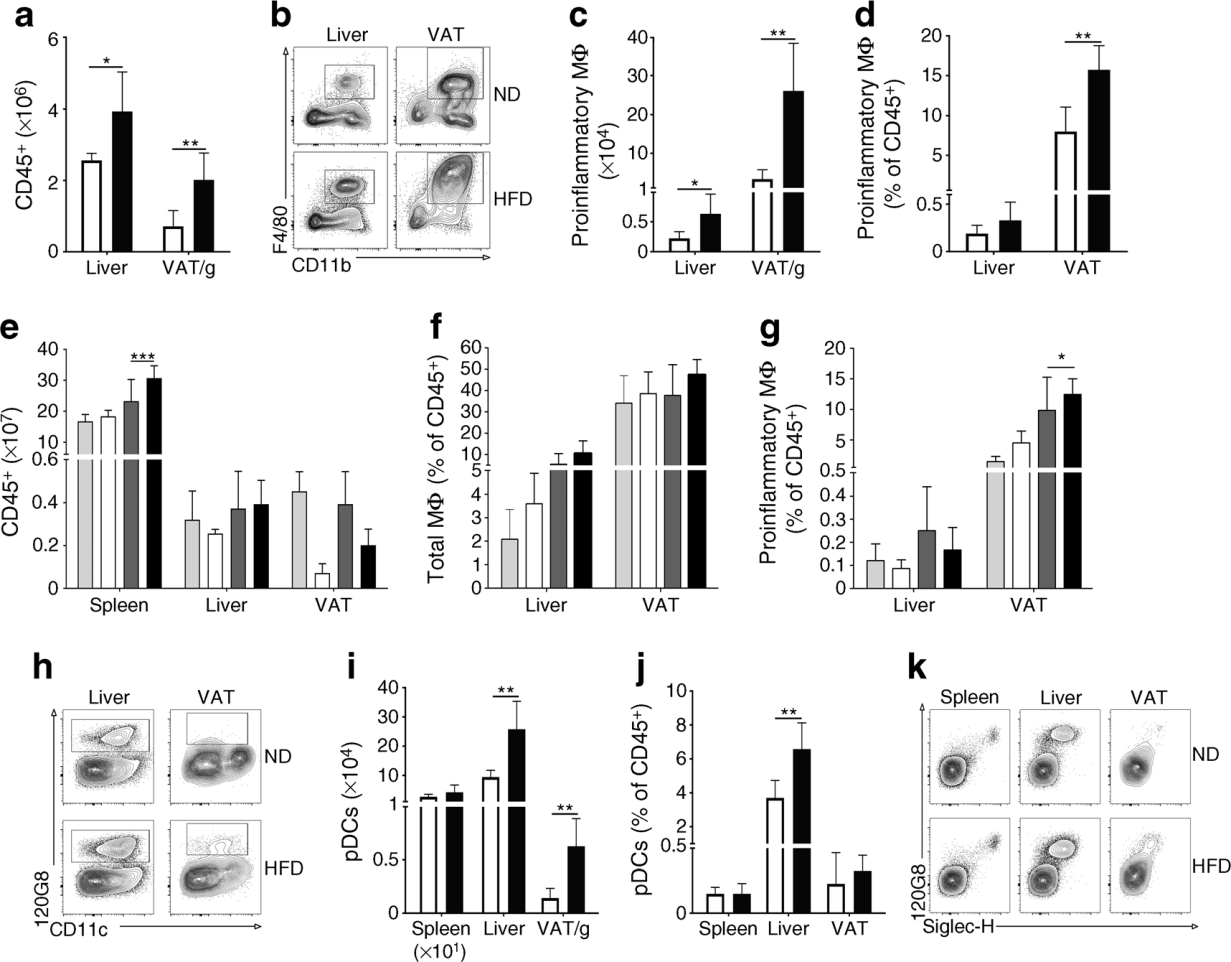
DIO promotes proinflammatory macrophage and pDC infiltration in the AT and liver. Flow cytometry analysis of leucocytes from the spleen and liver and SVF from the VAT of lean (white bars) and obese (black bars) B6 mice or lean (light grey bars) and obese (dark grey bars) IFNAR^−/−^ mice. (**a**) Number of live CD45^+^ cells. (**b**) Representative contour plots of FVD^−^CD45^+^CD11c^+^F4/80^+^CD11b^+^ cells from the liver or VAT of lean (ND) and obese (HFD) B6 mice with the gating for the proinflammatory macrophage subset of total CD45^+^ cells shown. (**c**) Number and (**d**) percentage of FVD^−^CD45^+^CD11c^+^F4/80^+^CD11b^+^ proinflammatory macrophages out of CD45^+^ cells. (**e**) Number of live CD45^+^ cells. (**f**) Percentage of total FVD^−^CD45^+^CD11b^+^F4/80^+^ macrophages out of live CD45^+^ cells. (**g**) Percentage of FVD^−^CD45^+^CD11c^+^F4/80^+^CD11b^+^ proinflammatory macrophages out of live CD45^+^ cells. (**h**) Representative contour plots of FVD^−^CD45^+^CD19^−^B220^+^120G8^+^CD11c^int^ cells from the liver or VAT of lean (ND) and obese (HFD) B6 mice with the gating for the pDC subset of total live CD45^+^ cells shown. (**i**) Number and (**j**) percentage of pDCs out of live CD45^+^ cells. (**k**) Representative contour plots of FVD^−^CD45^+^CD19^−^B220^+^CD11c^int^ cells showing the expression of Siglec-H and 120G8. *n* = 6 in all groups of animals. The experiments were repeated independently at least 2 times. *p ≤ 0.05, **p ≤ 0.01, ***p ≤ 0.001 using Student’s *t* test. MΦ, macrophage

Obese mice developed a low-grade inflammation in the visceral fat deposits, with an increased number of leucocytes and an altered immune cell repertoire characterised by elevated levels of CD11c^+^ proinflammatory macrophages (CD11c^+^CD11b^+^F4/80^+^) (Fig. 2a–d).

When examining the infiltrating cell population in the inflamed AT, we found an expectedly large proportion of the CD11c^+^ cells to be proinflammatory macrophages (F4/80^+^CD11b^+^CD11c^+^) (Fig. 2b–d). In the liver the number of proinflammatory macrophages was also found to be significantly increased (Fig. 2c). When applying a subsequent analysis of the total CD11c^+^ population in the liver, we found significantly higher numbers in the obese state but also noted that a large proportion of these cells could not be accounted for by the macrophage population (Fig. 2c).

To determine whether the metabolic effects observed in the absence of type I IFN signalling were linked to a decreased inflammation in VAT and the liver we exposed the IFNAR^−/−^ mice and B6 control mice to an 18 week period of HFD feeding and compared them to groups fed an ND (Fig. 2e–g). We observed that the accumulation of proinflammatory macrophages in VAT of B6 mice was reduced in the absence of IFNAR1 (Fig. 2g). This would be in line with a previously suggested role of IFN-I in proinflammatory polarisation of VAT-recruited macrophages [22].

### DIO led to elevated numbers of pDCs

Because it has been previously reported that pDCs increase in both the liver and AT of HFD-treated animals [4], we hypothesised that this DC subset could account for the observed increase in CD11c^+^ cells in the liver. We therefore identified the pDCs among the CD11c^+^ population by gating away B cells (CD19^+^), natural killer cells (NK1.1^+^) and macrophages (F4/80^+^ and CD11b^+^) and focusing on the B220^+^120G8^+^CD11c^(int)^ subset (Fig. 2h–j). While the expression of the pDC marker Siglec-H was high in the splenic pDC population, the same subset in the liver and fat depots demonstrated variable expression profiles (Fig. 2k); the marker itself was also recently found to be less specific than initially proposed [25] and is partly sensitive to collagenase treatment [26]. The 120G8 antibody specifically targets BST-2 and constitutes a suitable marker for pDCs, as illustrated by its ability to eliminate pDCs through antibody depletion without significantly affecting other cell populations [27]. Using this gating strategy, we found that the pDC population was significantly increased in both the liver and VAT after HFD feeding (a 2.7-fold increase in the liver and 4.3-fold increase in VAT), and was most abundant in the liver (Fig. 2j). In the liver, pDCs accounted for almost 50% of the CD11c^+^ population in the obese group and approximately 30% in the lean group (Fig. 2h–j).

### pDC deficiency protects against weight gain

**Fig. 3:**
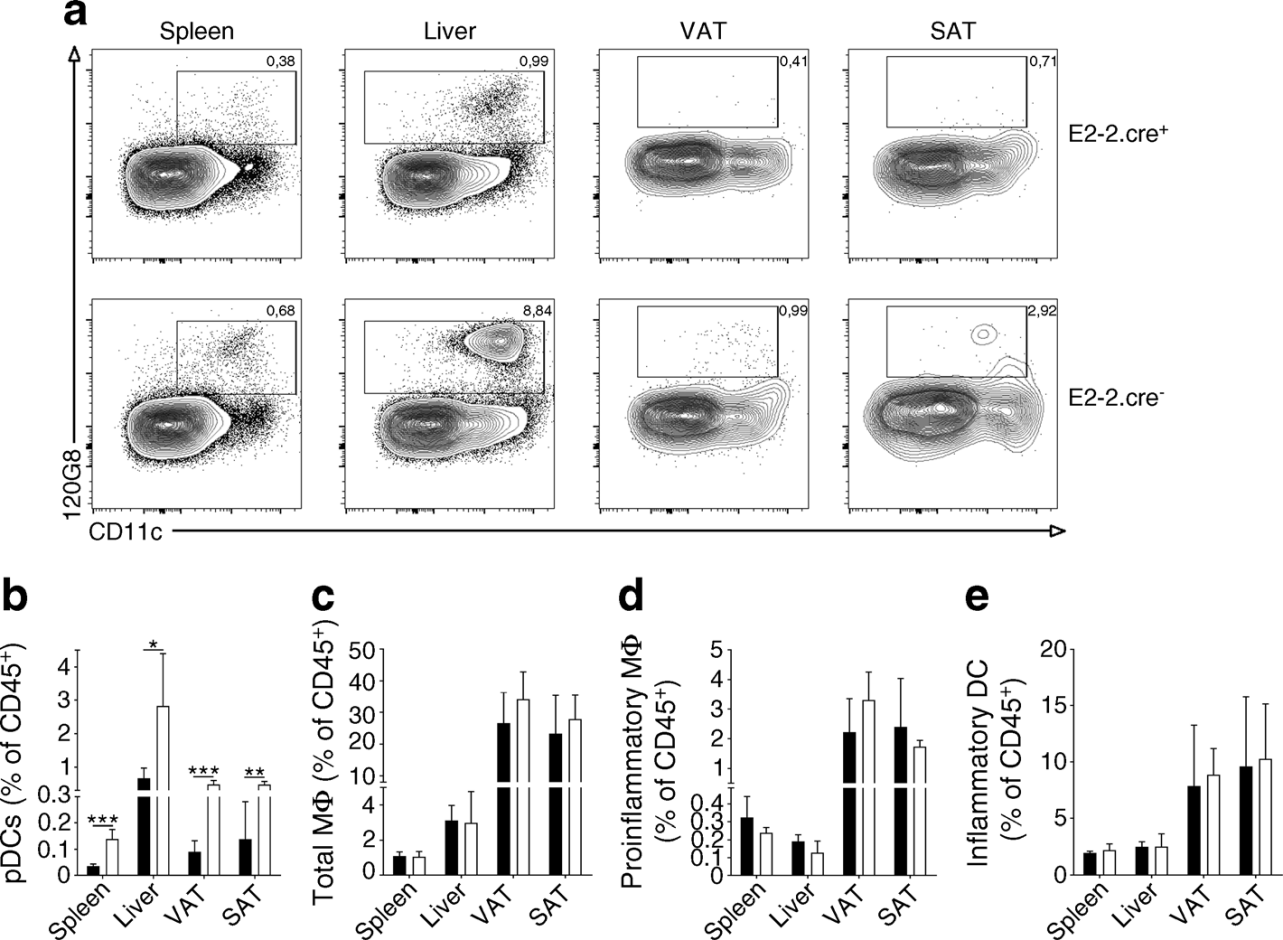
Significant reduction in pDCs in the E2-2.cre^+^ mouse. Flow cytometry analysis of CD45^+^ leukocytes from the spleen and liver and SVF from the VAT and SAT of E2-2.cre^+^ and E2-2.cre^−^ mice. (**a**) Representative contour plots of FVD^−^CD45^+^CD19^−^B220^+^120G8^+^CD11c^int^ cells with gating for pDCs. Percentages of (**b**) pDCs and (**c**) total FVD^−^CD45^+^CD11b^+^F4/80^+^ macrophages. (**d**) FVD^−^CD45^+^CD11c^+^F4/80^+^CD11b^+^ proinflammatory macrophages. (**e**) FVD^−^CD45^+^CD11c^hi^CD11b^−^MHCII^+^ DCs and in the spleen, liver, VAT and SAT of E2-2.cre^+^ (black bars) and E2-2.cre^−^ (white bars) mice. (**b**–**e**). *n* = 6 in each mouse group. The experiments were repeated independently at least 2 times. *p ≤ 0.05, **p ≤ 0.01, ***p ≤ 0.001 using Student’s *t* test. MΦ, macrophage

The observed accumulation of pDCs in both the liver and AT, as well as the protection from obesity development in the IFNAR knockout mice, suggest that this cellular subset could play a role in the pathology observed in the DIO model. To directly address this issue, we used the B6.E2-2^*fl*/*fl*^.Itgax-cre mouse strain, in which the conditional knockout of E2-2 results in a specific block of the development of pDCs [23] (Fig. 3). A pronounced decrease in this subset is evident in both the bone marrow [23] and peripheral organs, including the spleen, liver and AT (Fig. 3a, b). In contrast, no significant alteration was seen in other haematopoietic subsets, such as macrophages and DCs (Fig. 3c–e).

**Fig. 4:**
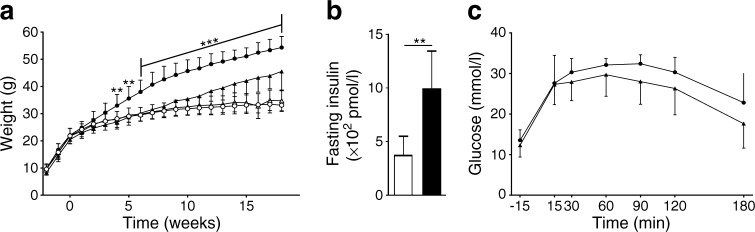
Characteristics and metabolic profile of E2-2.cre^+^ and E2-2.cre^−^ mice on ND or HFD. Mice were fed either an ND or HFD for 18 weeks, starting at 5 weeks of age. (**a**) Weekly weight measurements of the four groups of mice (*n* = 8 for ND and *n* = 12 for HFD): E2-2.cre^+^ND (white triangles) E2-2.cre^+^ HFD (black triangles), E2-2.cre^−^ ND (white circles), E2-2.cre^−^ HFD (black circles). (**b**) Fasting insulin levels for HFD-fed E2-2.cre^+^ (white bar) and E2-2.cre^−^ (black bar) mice. (**c**) An OGTT performed after 18 weeks (*n* = 6). Mice were given an oral dose of glucose (2 g/kg) and blood glucose was determined from tail vein samples at different time points. E2-2.cre^+^ HFD (black triangles), E2-2.cre^−^ HFD (black circles). The experiments were repeated independently 2–3 times. **p ≤ 0.01, ***p ≤ 0.001 using two-way ANOVA (**a**, **c**) or Student’s *t* test (**b**)

Next we exposed E2-2 conditional knockout (E2-2.cre^+^) mice and control littermates (E2-2.cre^−^) to an 18 week period of HFD feeding and compared them to groups fed an ND. As with the IFNAR^−/−^ mice, we observed a significant difference in weight gain between the E2-2.cre^+^ and the E2-2.cre^−^ groups, with a significantly lower weight gain in the E2-2.cre^+^ mice (45.6 g for E2-2.cre^+^ and 54.4 g for E2-2.cre^−^ mice) that was evident from as early as week 4 on an HFD (Fig. 4a). The reduced weight gain was accompanied by increased insulin sensitivity (Fig. 4b). Thus, while the control group at the endpoint had reached the level of insulin resistance, the E2-2.cre^+^ mice were protected from developing insulin resistance (Fig. 4b). An improved, albeit not significant, GTT was also noted (Fig. 4c).

### Interruption of type I IFN signalling and pDC deficiency prevents DIO and insulin resistance by affecting energy metabolism

**Fig. 5:**
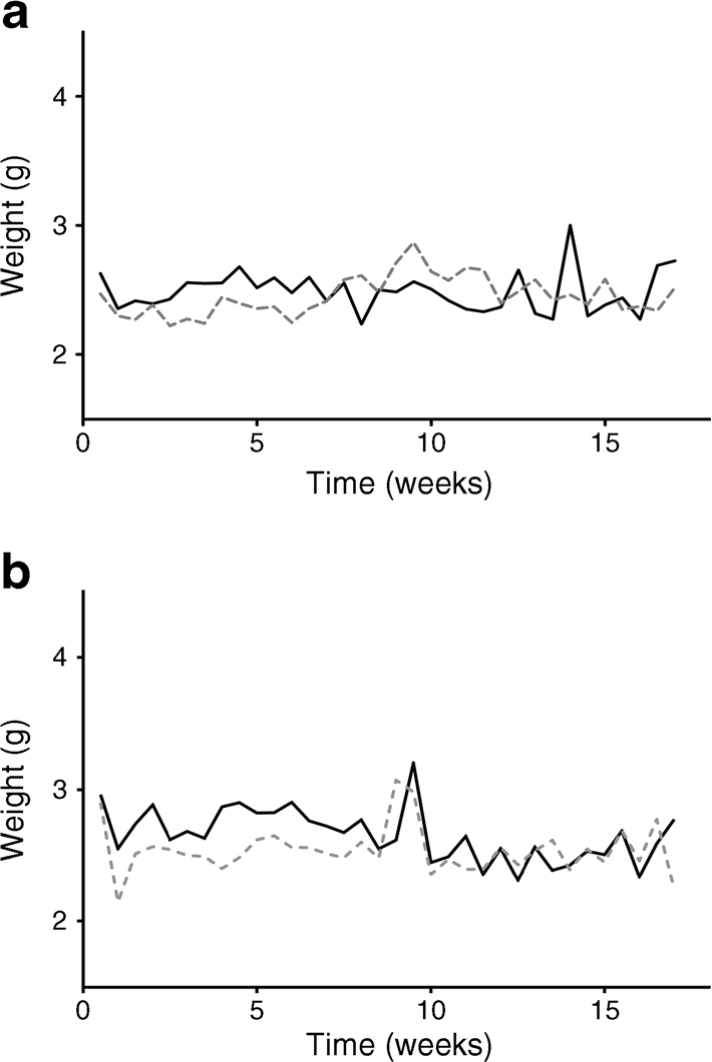
Food intake of E2-2.cre^+^ and IFNAR^−/−^ mice is unaltered compared with control mice. Food intake per cage for (**a**) IFNAR^−/−^ (dashed grey line) and B6 mice (black line) and (**b**) E2-2.cre^+^ (dashed grey line) and E2-2.cre^−^ (black line) littermates. *n* = 2–6 cages, *n* = 6 animals per cage. Two-way ANOVA was used; no significant differences were observed

To investigate if the observed protection from weight gain among the IFNAR^−/−^ mice was the result of a decrease in energy intake or energy expenditure, we registered the food intake throughout the experiment. As illustrated in Fig. 5a, no significant difference between the IFNAR^−/−^ and B6 mice was observed at any time point, suggesting that the observed difference in weight gain was due to different energy expenditure. Similarly, when the metabolic differences between E2-2.cre^+^ and E2-2.cre^−^ mice were assessed through their food intake, no significant difference was observed (Fig. 5b). Additionally, when assessing the mice, which were kept in groups of 4–6 individuals, no differences in behavioural patterns were seen between the groups. These results suggest that the ablation of type I IFN signalling, as well as pDC deficiency, prevented the mice from developing DIO and insulin resistance by affecting energy metabolism.

## Discussion

In the current study, we present evidence that type I IFN plays a critical role in the development of obesity and diabetes in the DIO mouse model of type 2 diabetes. Using IFNAR^−/−^ mice, we demonstrated that the absence of type I IFN signalling protected them from the development of DIO and diabetes. Since pDCs, a major source of type I IFN [16, 28], have been reported to accumulate in the AT and liver of obese mice [4], we reasoned that this cellular subset could constitute a key component in the type I IFN-mediated infiltration of proinflammatory M1 macrophages during obesity development. In agreement with this hypothesis, we demonstrated that pDC-deficient E2-2.cre^+^ mice displayed a similar resistance to developing DIO and insulin resistance as observed in IFNAR^−/−^ mice.

Type I IFNs are prominent in viral infections, where they promote host defence mechanisms. However, a role of the pleiotropic cytokine family in several biological processes has also been proposed [8, 11]. Type I IFNs have previously been indirectly implicated in obesity development based on the analysis of the IRF-7^−/−^ mouse [19]. IRF-7 plays an important role in innate immunity through the regulation of IFN-α/β secretion. These IFNs can further activate cells through interactions with IFNAR, thereby inducing additional IRF-7 expression, which is required for the type I IFN positive feedback loop. A study by Wang et al [19] suggests that improved energy expenditure protects IRF-7^−/−^ mice from developing DIO. In agreement with their findings, our data suggest that the protection from weight gain in the absence of type I IFN signalling is a consequence of altered immune–metabolism interplay, resulting in increased energy metabolism. This is in line with the notion that the immune system may play an important role in maintaining energy balance.

The pDCs are professional type I IFN producers responsible for the vast majority of IFN-α/β secretion [29]. While pDCs are most well-known for their antiviral activity and ability to secrete vast amounts of IFN-I in response to TLR recognition of double-stranded RNA and CpG motifs, these cells have also been implicated in several disease conditions associated with systemic inflammation, including autoimmune diabetes [30, 31]. An abundance of nucleic acid ligands and a critical role of TLR-9 in obesity-associated inflammation has also been reported previously both in animal models and humans [22, 32–35]. The finding that IFNAR^−/−^ mice are protected from DIO highlights a possible role of pDCs in obesity-associated inflammation. While the pDCs have previously been found at an elevated frequency in the AT and liver of obese mice [4], human studies have found decreased [36–38] or unaltered [30, 39] numbers of circulating pDCs in obese individuals with type 2 diabetes compared with lean individuals but have been unable to investigate the possible relocation of these cells. The data obtained from the analysis of the mouse model of DIO reported here and previously by Stefanovic-Racic et al [4] supports the notion of a recruitment and activation of pDCs in obese VAT possibly, as has been previously suggested, mediated by the adipokine chemerin [22, 40]. However, in light of conflicting results [6], further studies are needed in order to elucidate this possibility.

The accumulation of pDCs in the liver during obesity development may suggest a role of this subset in this organ. Similar to the AT, the accumulation of pDCs in the liver was correlated with an increase in the number of proinflammatory macrophages. Even at steady state, pDCs are highly abundant in the liver, accounting for one-third of the DC population. While the precise reason for this accumulation is unknown, it is likely to be part of the requirement for the optimal defence mechanisms of the liver, as it encounters an array of substances through processed blood from the gastrointestinal tract. This includes gut-derived commensal bacterial products [41, 42], of which the composition is affected by what is ingested. As pDCs are known to play an important role in the defence against invading microbes, it is plausible that even a swift change in the nutrient composition could induce their TLR-mediated production of proinflammatory cytokines [43]. Indeed, studies have shown that the gut microbiome rapidly responds to dietary changes in both humans [44] and mice [45, 46], which has been proposed to contribute to obesity.

To directly address the pDCs as possible mediators of obesity development through their type I IFN-producing ability, we used a mouse with a conditional knockout of E2-2, the E2-2.cre^+^ mouse, resulting in a specific block in the development of pDCs [23]. We found that these mice were protected from DIO development, comparable to the IFNAR^−/−^ mice. In addition, the mice were also protected from developing insulin resistance and showed mildly improved glucose tolerance. Taken together, our results suggest a key role for pDC-derived type I IFN in obesity development. This concurs with, and gives genetic evidence for, a model recently presented by Ghosh et al [22] suggesting that low-grade inflammation associated with obesity is driven by type 1 IFN produced by pDCs recruited to VAT and leading to proinflammatory polarisation of adipose-resident macrophages.

## Abbreviations

AT: Adipose tissue
BAT: Brown adipose tissue
DC: Dendritic cell
DIO: Diet-induced obesity
FVD: Fixable viability dye
HFD: High-fat diet
IFNAR: IFN-α receptor
MCP-1: Monocyte chemoattractant protein-1
ND: Normal diet
pDC: Plasmacytoid dendritic cell
SAT: Subcutaneous adipose tissue
SVF: Stromal vascular fraction
TLR: Toll-like receptor
VAT: Visceral adipose tissue
